# *Bifidobacterium* supplementation maintains gut microbiota stability and enhances well-being during short-term travel

**DOI:** 10.3389/fnut.2026.1724829

**Published:** 2026-02-12

**Authors:** Long Wang, Yue Yu, Xiaxian Shen, Xiaohui Li, Danni Wang, Yue Zhai, Wei Jiang, Wei Zhao, Qinghua Yu, Min-Tze Liong, Dongbo Chen, Ai Zhao

**Affiliations:** 1 Tongji University Affiliated Shanghai Pulmonary Hospital, Shanghai, China; 2 Laboratory of Microbiology, Immunology, and Metabolism, DiPROBIO (Shanghai) Co., Ltd., Shanghai, China; 3 DiPRO Microecological Health Management Center, Shanghai, China; 4 Vanke School of Public Health, Tsinghua University, Beijing, China

**Keywords:** antibiotic resistant genes, *Bifidobacterium*, gut microbiota, probiotic, short term travel

## Abstract

**Background:**

International travel exposes individuals to abrupt environmental, dietary, and circadian changes that can disturb gut microbiota and overall well-being. While probiotics are known to support gastrointestinal and systemic health, their effects during short-term travel remain incompletely characterized in randomized trials.

**Methods:**

This randomized, double-blind, placebo-controlled study investigated whether a multi-strain *Bifidobacterium* probiotic could maintain gut microbiota stability and support health during a five-day trip from China to Japan. Forty healthy adults were randomly assigned to receive either probiotic (*n* = 22) or placebo (*n* = 18) daily from Day 1 to Day 4. Stool samples collected before departure (Day 0) and after return (Day 5) were analyzed by metagenomic sequencing, quantitative PCR, and fecal secretory immunoglobulin A (sIgA) assays. Participants completed validated questionnaires on gastrointestinal and respiratory symptoms, sleep quality (PSQI), anxiety (GAD-7), and well-being (WHO-5).

**Results:**

Compared with placebo, participants receiving the probiotic showed maintenance of microbial diversity (Chao1 and Fisher indices, both *p* = 0.044), prevented enrichment of potentially harmful taxa (*Bilophila*, *Flavonifractor*), and increased *Bifidobacterium* abundance. Clinically, the probiotic group reported fewer respiratory and systemic symptoms, including sore throat (*p* = 0.034) and fatigue (*p* = 0.043). Sleep quality also improved, with longer sleep duration (*p* = 0.023), fewer total occurrence days of PSQI >5 (*p* = 0.009), lower anxiety scores (*p* = 0.001) and higher WHO-5 well-being scores (*p* = 0.041). Functional profiling showed up-regulation of vitamin biosynthesis pathways (folate, biotin, retinol) and decreased antibiotic resistance gene prevalence.

**Conclusion:**

Short-term probiotic administration demonstrated gut microbiota resilience and improved physiological and psychological stability during travel. Probiotics may serve as an accessible strategy to support well-being under transient environmental and lifestyle stress.

**Clinical trial registration:**

ClinicalTrials.gov, identifier NCT07163819.

## 1 Introduction

International travel is often accompanied by abrupt changes in diet, environment, water sources, and circadian rhythm. These rapid transitions can disrupt intestinal microbial communities, leading to transient dysbiosis and gastrointestinal discomfort (1). Evidence from metagenomic and longitudinal cohort studies has demonstrated that even brief exposure to new environments can significantly alter gut microbial composition, sometimes within days (2). Such microbial fluctuations may also influence immune, metabolic, and psychological states through the microbiota–gut–brain axis (3).

Short-term international travel represents a unique and clinically relevant stress model for studying gut microbiota stability. Unlike chronic lifestyle or disease-related perturbations, travel-associated stressors are acute, multifactorial, and time-limited, typically combining abrupt dietary shifts, circadian disruption, environmental microbial exposure, and psychosocial stress within a well-defined period. This makes short-term travel particularly suitable for controlled interventional studies aimed at assessing resilience and recovery of the gut microbiome.

Traveler’s diarrhea (TD) remains the most common illness associated with travel, affecting up to 70% of individuals depending on region and season. However, travel-related discomfort extends beyond diarrhea to include abdominal pain, bloating, constipation, or irregular bowel habits—symptoms that can impair overall well-being and productivity (4). Concurrently, international mobility contributes to the global spread of antimicrobial-resistant bacteria and resistance genes (5, 6). The intestinal microbiota acts as a reservoir for these elements, and studies have shown that antibiotic resistance gene (ARG) abundance often increases after foreign travel (7–9).

Sleep and mood disturbances are additional concerns during travel, arising from time zone shifts, altered routines, and environmental stress (10). Disrupted sleep can influence gut microbiota composition, while microbial dysbiosis may in turn affect neurotransmitter balance, inflammation, and stress response—demonstrating a bidirectional link between gut and brain function (11–13). The potential implications of this microbiome-gut-brain axis for travelers are significant. Disruptions to sleep patterns often lead to exacerbated gastrointestinal issues, which are common complaints among travelers. These issues can range from mild discomfort to more severe conditions such as irritable bowel syndrome or inflammatory bowel disease (14, 15).

Probiotics, defined as “live microorganisms that confer health benefits to the host when administered in adequate amounts (16),” offer various health protections ranging from immunity, anti-inflammatory and inhibition of pathogens (17, 18). Among probiotic taxa, *Bifidobacterium* species are of particular mechanistic relevance in the context of travel-related stress. They play a central role in complex carbohydrate fermentation, vitamin biosynthesis, maintenance of intestinal barrier integrity, and modulation of mucosal immune responses. In addition, *Bifidobacterium* species contribute to colonization resistance against opportunistic and exogenous microbes, a function that may be especially important during rapid environmental transitions (19). Several clinical studies have reported that probiotics improve bowel regularity, enhance immune response, and modestly reduce the risk of traveler’s diarrhea (20). Moreover, probiotics have been shown to improve psychological well-being through modulation of the gut–brain axis (21, 22).

Despite extensive probiotic research, randomized controlled evidence specifically addressing whether targeted probiotic supplementation can preserve gut microbiota stability and host well-being during short-term international travel remains scarce. Previous studies often lacked standardized travel destinations, consistent diets, or precise intervention timing (23, 24). As a result, the capacity of probiotics to buffer acute, real-world environmental stressors over a short time frame has not been systematically evaluated. A short-term, destination-controlled model provides a valuable framework for understanding how probiotics buffer environmental stress within a few days.

The present randomized, double-blind, placebo-controlled trial therefore aimed to evaluate whether a multi-strain *Bifidobacterium* probiotic could (i) prevent travel-induced perturbations of gut microbiota, (ii) reduce gastrointestinal and respiratory symptoms, (iii) support immune and vitamin-related metabolic functions, and (iv) improve sleep and psychological well-being during short-term international travel.

## 2 Methods

### 2.1 Study design and ethics approval

This randomized, double-blind, placebo-controlled clinical trial was conducted according to the Declaration of Helsinki and CONSORT 2010 guidelines. Participants were randomly assigned in a 1:1 ratio to probiotic or placebo groups using a computer-generated sequence by an independent statistician who was not involved in participant recruitment, intervention administration, or outcome assessment.

Allocation concealment was ensured by using sequentially numbered, identical containers that were labeled according to the randomization code and distributed by study personnel who had no access to the allocation list. Both participants and investigators remained blinded to group assignment throughout the intervention and outcome assessment period, as the probiotic and placebo products were identical in appearance, packaging, and labeling. Investigators, caregivers, participants, laboratory staff, data managers, and statisticians were fully blinded to group allocation throughout the trial, and the randomization code was not broken until all clinical and microbiome analyses were completed.

The study protocol was approved by the Tsinghua University Science and Technology Ethics Committee (Approval No. THU-01-2025-1007), and registered at ClinicalTrials.gov (Registration No. NCT07163819). Written informed consent was obtained from all participants prior to enrolment.

### 2.2 Participants and sample size

Healthy adults aged 18–65 years planning a standardized five-day round trip to Japan were recruited. Exclusion criteria included the use of antibiotics, probiotics, hormones, or immunosuppressants within 4 weeks before enrolment; chronic systemic disease; major surgery within the previous month; or known allergy to probiotic components. Sample size was calculated based on expected differences in gut microbiota diversity (Cohen’s *d* = 1.0), requiring 18 participants per arm for 85% power at *α* = 0.05. Allowing for 20% attrition, 46 participants were enrolled.

### 2.3 Intervention

Participants received either a probiotic or placebo product which was provided as an oil-based suspension in a dropper bottle. The active formulation consisted of 90% sunflower oil and 10% probiotic powder, delivering a total of 1.5 × 10^9^ CFU per daily serving (6 drops), with each of the three strains, *Bifidobacterium longum* subsp. *infantis* M-63, *B. breve* M-16V, and *B. longum* BB536, contributing 0.5 × 10^9^ CFU. The matching placebo was identical in appearance and formulation, containing 90% sunflower oil and 10% maltodextrin, but without any probiotic strains. Participants consumed six drops daily from Day 1 to Day 4 of travel. Compliance was verified by diary logs and returned packaging.

### 2.4 Questionnaires

Validated instruments were used to evaluate health outcomes: the Pittsburgh Sleep Quality Index (PSQI) (25), Generalized Anxiety Disorder Scale (GAD-7) (26), and World Health Organization Five Well-Being Index (WHO-5) (27). Participants recorded daily gastrointestinal and respiratory symptoms and completed standardized questionnaires before departure, during travel, and after return.

### 2.5 Fecal sample collection

Two stool samples (~5 g each) were collected from each participant: pre-travel (Day 0) and post-travel (Day 5). Samples were stored at 4 °C, transported to the laboratory within 3 days, and frozen at −80 °C until analysis.

### 2.6 DNA extraction and metagenomic sequencing

DNA was extracted using the NucleoSpin 96 Soil Kit (Macherey-Nagel, Düren, Germany) with bead-beating for cell lysis. DNA purity was verified by Qubit 2.0 fluorometry (Thermo Fisher Scientific, Waltham, MA, United States). Libraries were prepared using the NEBNext Ultra II DNA Library Prep Kit (New England Biolabs, Ipswich, MA, United States) and sequenced using a paired-end strategy (2 × 150 bp) with an average sequencing depth of 6 Gb per sample on an Illumina NovaSeq 6000 platform (Illumina, San Diego, CA, United States). Reads were trimmed (Trimmomatic v0.36), human sequences filtered (Bowtie2 v2.5), assemblies generated (MEGAHIT v1.2), and genes predicted (Prodigal v2.6). Taxonomic classification used Kraken2 (version 2.1.2) with the RefSeq database (release 214, downloaded on 02/10/2025), and functional annotation was performed via HUMAnN3 with KEGG mapping.

### 2.7 LEfSe and KEGG pathway analysis

Differential taxa were identified using LEfSe with *α* = 0.05 (Kruskal–Wallis test) and LDA ≥2.0. KEGG pathways were annotated using the KAAS bi-directional best-hit method, and differential abundances analyzed in STAMP v2.1.

### 2.8 Quantitative PCR and sIgA

Species-specific qPCR assays quantified *Bifidobacterium* taxa using multiplex primers on an ABI 7500 system (Applied Biosystems, Foster City, CA, United States). Fecal secretory immunoglobulin A (sIgA) was measured by ELISA (BBI Life Sciences, Shanghai, China; D711176). Data were expressed as log₁₀ gene copies/g feces and μg/g feces, respectively.

The primer sets used were:

**Table:** 

Target species	Forward primer (5′ → 3′)	Reverse primer (5′ → 3′)	Probe (5′ → 3′)
*B. adolescentis*	GCTGATATCTGCGCTGTACC	AAACCACCCAGTAGTCCTCC	5′ROX-AATCATGACCAATGCCGCACC-3′BHQ2
*B. animalis* subsp. *lactis*	ACCTCACCAATCCGCTGTTC	GATCCGCATGGTGGAACTCT	5′VIC-CGGTTTGCCCTCGTTGTCCGTGGC-3′BHQ1
*B. bifidum*	CTGGCAGCCGTGACACTACT	TGAACTGGCCGTTACGGTCT	5′CY5-CGTGACGCCGGTCAGCGACA-3′BHQ3
*B. breve*	AATCTGAGTGAGCGGTTGCC	GCATGACCGTCAAGTGTGGC	5′CY5-AACGTCATCACGGCAAGGT-3′MGB
*B. longum* subsp. *infantis*	CGGTCTTCTACAGGAAGCGG	CCACGCTTTCCTCGTCCATA	5′FAM-ACGACTTATGATCGATCGCG-3′MGB
*B. longum* subsp. *longum*	ACCAAGTTCCAGCCCACAGC	CGCCATACCAGTAGTAGGCG	5′VIC-ACCGTGCGCTTGGATGTGT-3′MGB
*B. pseudocatenulatum*	GCTTCCCTCGTATGATGGGTT	TTTCCACGCGTGCTTCTTTC	5′ROX-TGCAGATCAACTGCGCCGCG-3′BHQ2
General *Bifidobacterium*	GTCCGGTGTGAAAGTCCATC	GTAACGGCCCAGAGACCT	5′FAM-TCGCCATTGGTGTTCTTCCCG-3′DAB

Standard curves were constructed using serial dilutions of plasmids containing cloned target sequences, and results were expressed as log_10_ gene copies per gram of wet feces.

### 2.9 Antibiotic resistance genes

Antibiotic resistance genes (ARGs) (*blaSHV*, *mefE*, *tetA*) were detected by PCR as a semi-quantitative approach, with products visualized via 2% agarose electrophoresis. Band intensity was quantified using Image Lab software.

The primer sets used were:

**Table:** 

Target gene	Primer sequence (5′ → 3′)	Amplicon size (bp)	Annealing temperature	Cycles
*bla*SHV	F: AGCCGCTTGAGCAAATTAAAC	710	57 °C	35
	R: ATCCCGCAGATAAATCACCAC			
*mef*E	F: GGGAGATGAAAAGAAGGAGT	364	57 °C	35
	R: TAAAATGGCACCGAAAG			
*tet*A	F: CGGTCTTCTTCATCATGCAAC	83	57 °C	35
	R: GTCCCAGTGAAAGCGATCC			

### 2.10 Statistical analysis

Analyses were conducted in R v4.3.2. Alpha diversity indices (Chao1, Fisher) were calculated using the *vegan* package; beta diversity was assessed with Bray–Curtis dissimilarity (PERMANOVA, 999 permutations). Integration, pre-processing and harmonization of metagenomic sequencing data for taxonomic profiling, KEGG, and LEfSe were conducted using Gi-MAPS (DiproX, Shanghai, China) prior to downstream statistical analyses. Continuous variables were analyzed using Mann–Whitney *U* tests, and categorical data by *χ*^2^, Fisher or McNemar’s test. *p* < 0.05 was considered significant.

To support interpretation of clinical relevance beyond statistical significance, analyses of validated questionnaires incorporated established clinical cut-offs and severity categories. For sleep quality, a global PSQI score >5 was used to define clinically relevant poor sleep quality, as established in the original PSQI validation and widely applied in clinical and epidemiological studies (25). Changes in the proportion of participants or days exceeding this threshold were considered indicative of clinically meaningful alterations in sleep quality. The GAD-7 score further categorized symptoms as minimal (0–4), mild (5–9), moderate (10–14), or severe (≥15). Scores <5 were considered within the non-clinical range, reflecting the absence of clinically significant anxiety (26). Accordingly, between-group differences within this range were interpreted as changes in stress sensitivity and emotional resilience rather than treatment of an anxiety disorder. Based on prior validation studies, a relative change of ≥10% in WHO-5 score was considered clinically meaningful, representing a perceptible improvement in subjective well-being and daily functioning rather than random variation (28).

## 3 Results

### 3.1 Participant flow and baseline characteristics

**Figure 1:**
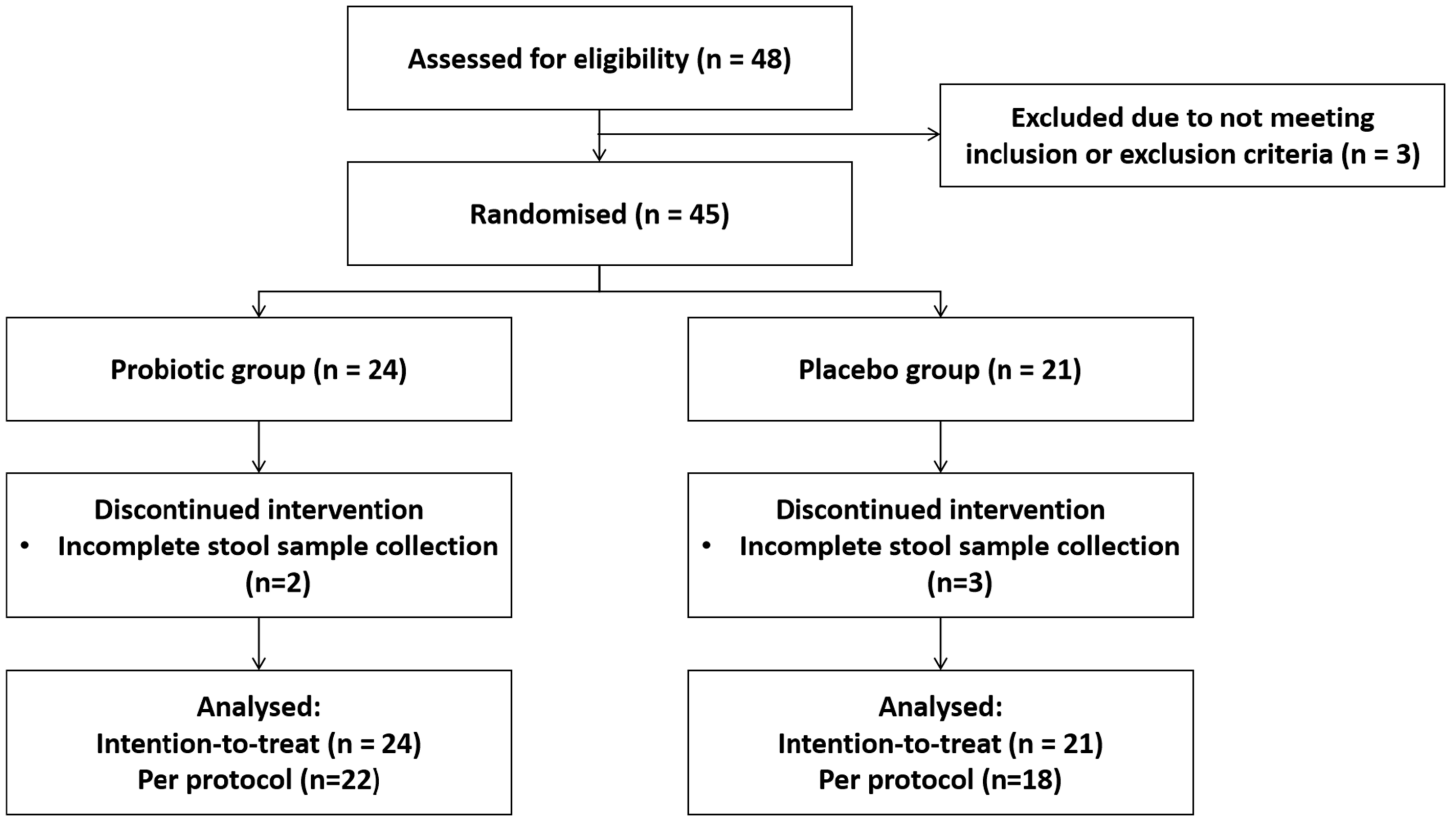
CONSORT flow of participant screening, randomization, allocation, and analysis inclusion. Of 48 screened individuals, 45 were randomized and 40 included in final analysis (22 probiotic, 18 placebo). No withdrawals or adverse events occurred, confirming good compliance. The study maintained high adherence and data completeness across participants. CONSORT, Consolidated Standards of Reporting Trials.

Of the 48 individuals screened, 45 met eligibility criteria and were randomized into probiotic (*n* = 24) or placebo (*n* = 21) groups. Five participants (two probiotic, three placebo) were excluded due to incomplete stool sample collection, resulting in 22 and 18 participants per group, respectively, for per-protocol analysis (Figure 1). Baseline demographic characteristics, including age, gender, BMI, and lifestyle habits, did not differ significantly between groups (Table 1). No participant reported adverse events or use of additional probiotics, antibiotics, or medications during the study, indicating full compliance and good tolerability. Overall, the probiotic and placebo groups were well matched at baseline, confirming successful randomization and providing a robust foundation for attributing post-travel differences to the intervention.

**Table 1:** Baseline demographic and clinical characteristics of participants in the probiotic and placebo groups.

Variable	Placebo (*n* = 18)	Probiotic (*n* = 22)	*p*-value
Sex			0.145
Male	8 (44.4)	5 (22.7)	
Female	10 (55.6)	17 (77.3)	
Age, years	38.31 ± 6.33	36.91 ± 5.94	0.476
Height, cm	167.17 ± 8.77	163.41 ± 6.91	0.138
Weight, kg	64.86 ± 14.62	61.68 ± 10.90	0.436
BMI	22.99 ± 3.80	23.07 ± 3.73	0.944
Marital status			0.471
Married	15 (83.3)	20 (90.9)	
Unmarried	3 (16.7)	2 (9.1)	
Education level			0.903
Junior high or below	3 (16.7)	3 (13.6)	
High school	2 (11.1)	4 (18.2)	
College diploma	5 (27.8)	8 (36.4)	
Bachelor	7 (38.9)	6 (27.3)	
Master or above	1 (5.6)	1 (4.5)	
Household income			0.346
3,001–6,000 RMB	1 (5.6)	3 (13.6)	
6,001–10,000 RMB	4 (22.2)	8 (36.4)	
≥10,001 RMB	13 (72.2)	11 (50.0)	
Smoking status			0.138
Yes	6 (33.3)	3 (13.6)	
No	12 (66.7)	19 (86.4)	
Self-rated health			0.079
Excellent	2 (11.1)	4 (18.2)	
Good	8 (44.4)	16 (72.7)	
Fair	7 (38.9)	2 (9.1)	
Poor	1 (5.6)	0 (0.0)	
Travel in past 3 months			0.897
Yes	7 (38.9)	9 (40.9)	
No	11 (61.1)	13 (59.1)	

Values are mean ± SD or *n* (%). There were no significant differences between groups at baseline, confirming successful randomization and comparability before intervention. Statistical comparisons were conducted using the Mann–Whitney *U* test or *χ*^2^ test. BMI, body mass index; SD, standard deviation; *p* < 0.05 considered significant.

### 3.2 Respiratory, gastrointestinal, and systemic symptoms

The probiotic group experienced a lower frequency of several travel-related symptoms compared with placebo (Table 2). Probiotic supplementation demonstrated a targeted beneficial effect, significantly reducing the incidence of sore throat (*p* = 0.034) and fatigue (*p* = 0.043) during travel. The prevalence of other measured respiratory, gastrointestinal, and systemic symptoms was comparable between the probiotic and control groups. This finding indicated that the primary symptomatic benefits of the intervention for short-term travelers are associated with upper respiratory and general fatigue. Taken together, these findings indicate that probiotic supplementation selectively reduced upper respiratory and systemic symptoms associated with travel-related stress, rather than broadly altering all reported symptoms.

**Table 2:** Prevalence of respiratory, gastrointestinal, and systemic symptoms during short-term travel.

	Baseline (Day 0)			Post travel (Day 5)			Total occurrence days (Days 1–4)		
Health symptoms	Placebo	Probiotic	*p*-value	Placebo	Probiotic	*p*-value	Placebo	Probiotic	*p*-value
Respiratory symptoms									
Cough	1 (5.56%)	0 (0%)	0.450	2 (11.11%)	2 (9.09%)	1.000	2 (11.11%)	2 (9.09%)	1.000
Runny nose	0 (0%)	2 (9.09%)	0.492	0 (0%)	0 (0%)	1.000	0 (0%)	0 (0%)	1.000
Nasal congestion	0 (0%)	1 (4.55%)	1.000	0 (0%)	1 (4.55%)	1.000	0 (0%)	1 (4.55%)	1.000
Fever (≥37.5 °C)	0 (0%)	0 (0%)	1.000	0 (0%)	0 (0%)	1.000	0 (0%)	0 (0%)	1.000
Wheezing	0 (0%)	0 (0%)	1.000	0 (0%)	0 (0%)	1.000	0 (0%)	0 (0%)	1.000
Sore throat	0 (0%)	0 (0%)	1.000	3 (16.67%)	0 (0%)	0.083	4 (22.22%)	0 (0%)	0.034
Sneezing	1 (5.56%)	1 (4.55%)	1.000	1 (5.56%)	0 (0%)	0.450	1 (5.56%)	0 (0%)	0.450
Hoarseness	0 (0%)	0 (0%)	1.000	1 (5.56%)	0 (0%)	0.450	1 (5.56%)	0 (0%)	0.450
Cough with sputum	0 (0%)	0 (0%)	1.000	0 (0%)	0 (0%)	1.000	0 (0%)	0 (0%)	1.000
Pain when swallowing	0 (0%)	0 (0%)	1.000	2 (11.11%)	0 (0%)	0.196	3 (16.67%)	0 (0%)	0.083
Body aches	2 (11.11%)	0 (0%)	0.196	0 (0%)	0 (0%)	1.000	0 (0%)	0 (0%)	1.000
Gastrointestinal symptoms									
Diarrhea	2 (11.11%)	3 (13.64%)	1.000	3 (16.67%)	1 (4.55%)	0.310	4 (22.22%)	6 (27.27%)	1.000
Increased stool frequency	1 (5.56%)	1 (4.55%)	1.000	2 (11.11%)	0 (0%)	0.196	5 (27.78%)	1 (4.55%)	0.073
Bloody stool	0 (0%)	0 (0%)	1.000	1 (5.56%)	0 (0%)	0.450	1 (5.56%)	0 (0%)	0.450
Abdominal pain	0 (0%)	0 (0%)	1.000	1 (5.56%)	0 (0%)	0.450	5 (27.78%)	1 (4.55%)	0.073
Abdominal cramping	0 (0%)	0 (0%)	1.000	0 (0%)	0 (0%)	1.000	0 (0%)	0 (0%)	1.000
Constipation	2 (11.11%)	2 (9.09%)	1.000	1 (5.56%)	2 (9.09%)	1.000	6 (33.33%)	5 (22.73%)	0.695
Belching/Bloating/Flatulence	2 (11.11%)	1 (4.55%)	0.579	1 (5.56%)	3 (13.64%)	0.613	4 (22.22%)	4 (18.18%)	1.000
Nausea/Vomiting	0 (0%)	0 (0%)	1.000	0 (0%)	0 (0%)	1.000	0 (0%)	0 (0%)	1.000
Acid reflux	1 (5.56%)	1 (4.55%)	1.000	0 (0%)	0 (0%)	1.000	0 (0%)	0 (0%)	1.000
Rectal pain	0 (0%)	0 (0%)	1.000	0 (0%)	0 (0%)	1.000	0 (0%)	0 (0%)	1.000
Decreased appetite	1 (5.56%)	0 (0%)	0.450	0 (0%)	0 (0%)	1.000	0 (0%)	0 (0%)	1.000
Food refusal	0 (0%)	0 (0%)	1.000	0 (0%)	0 (0%)	1.000	0 (0%)	0 (0%)	1.000
Other symptoms									
Irritability	4 (22.22%)	1 (4.55%)	0.155	3 (16.67%)	1 (4.55%)	0.310	4 (22.22%)	1 (4.55%)	0.155
Fatigue	8 (44.44%)	5 (22.73%)	0.307	11 (61.11%)	7 (31.82%)	0.125	14 (77.78%)	9 (40.91%)	0.043
Dizziness	4 (22.22%)	1 (4.55%)	0.147	2 (11.11%)	0 (0%)	0.196	3 (16.67%)	2 (9.09%)	0.642
Headache	2 (11.11%)	0 (0%)	0.184	1 (5.56%)	0 (0%)	0.435	3 (16.67%)	1 (4.55%)	0.310
Dehydration	0 (0%)	0 (0%)	1.000	1 (5.56%)	0 (0%)	0.450	1 (5.56%)	0 (0%)	0.450

Values are presented as *n* (%) of participants reporting each symptom at baseline (Day 0), post-travel (Day 5), and during travel (total occurrence days, Days 1–4). *χ*^2^ test or Fisher’s exact test was used for comparisons. *p* < 0.05 indicates statistical significance.

### 3.3 Stool consistency

Stool types, assessed using the Bristol Stool Scale, demonstrated favorable normalization in the probiotic group (Table 3). At baseline, stool consistency was comparable between groups. By post-travel (Day 5), the probiotic group had a significantly higher prevalence of Type 5 stools (*p* = 0.007) and a lower prevalence of Type 6 stools (*p* = 0.013) compared to the placebo group. A majority of probiotic participants maintained stool types in the 4–5 range, indicating more stable bowel patterns and a significant reduction in loose stools. Overall, probiotic supplementation promoted clinically favorable stool consistency and reduced the occurrence of loose stools, indicating improved bowel stability during short-term travel.

**Table 3:** Distribution of stool types in probiotic and placebo groups before, during, and after travel.

	Baseline (Day 0)			Post travel (Day 5)		
Stool type	Placebo	Probiotic	*p*-value	Placebo	Probiotic	*p*-value
Constipation (1–2)	3 (16.67%)	0 (0%)	0.128	2 (11.76%)	0 (0%)	<0.001
Normal (3–5)	13 (72.22%)	18 (81.82%)		7 (41.18%)	22 (100%)	
Loose (6–7)	2 (11.11%)	4 (18.18%)		8 (47.06%)	0 (0%)	
Stool type						
1	0 (0%)	0 (0%)	-	0 (0%)	0 (0%)	—
2	3 (16.67%)	0 (0%)	0.165	2 (11.11%)	0 (0%)	0.382
3	4 (22.22%)	7 (31.82%)	0.749	4 (22.22%)	5 (22.73%)	1.000
4	3 (16.67%)	9 (40.91%)	0.188	3 (16.67%)	8 (36.36%)	0.302
5	6 (33.33%)	2 (9.09%)	0.131	0 (0%)	9 (40.91%)	0.007
6	2 (11.11%)	4 (18.18%)	0.859	6 (33.33%)	0 (0%)	0.013
7	0 (0%)	0 (0%)	—	2 (11.11%)	0 (0%)	0.382

Values are *n* (%). Probiotic improved stool regularity, maintaining more normal stool forms (Types 4–5) and reducing loose stools (Type 6). Values indicate average stool type per participant at each timepoint. *χ*^2^ test used for comparisons. Type 1–2 = constipation; Type 3–5 = normal; Type 6–7 = loose; *p* < 0.05 significant.

### 3.4 Sleep, anxiety, and well-being

#### 3.4.1 Sleep

Sleep duration, measured by the Pittsburgh Sleep Quality Index (PSQI), improved significantly in the probiotic group (Table 4). Participants receiving probiotics reported longer sleep at post-travel (Day 5, *p* = 0.023) and across travel Days 1–4 (*p* = 0.034). Daily analysis indicated prolonged sleep for the probiotic group on Day 2 and Day 5 based on PSQI score and, Day 3 and Day 5 based on actual sleep hours (*p* < 0.05; Supplementary Figure 1). Furthermore, the proportion of days with poor sleep quality (PSQI >5) during travel was significantly lower in the probiotic group compared with placebo (52.3% vs. 73.6%, *p* = 0.009). This reduction indicated fewer episodes of clinically relevant sleep disturbance rather than isolated improvements in individual sleep parameters. While other PSQI parameters such as latency and efficiency were comparable between groups, our findings suggest that probiotic supplementation reduced the burden of poor sleep episodes and improved overall sleep stability during short-term travel.

**Table 4:** Sleep duration and global PSQI scores during the intervention.

Domain	Baseline (Day 0)			Post travel (Day 5)			Difference Day 0 and Day 5			During travel (average Days 1–4)		
	Placebo	Probiotic	*p*-value	Placebo	Probiotic	*p*-value	Placebo	Probiotic	*p*-value	Placebo	Probiotic	*p*-value
Item-1: Subjective sleep quality	1.25 ± 0.20	0.91 ± 0.13	0.168	1.28 ± 0.18	0.86 ± 0.14	0.073	0.06 ± 0.27	−0.05 ± 0.19	0.764	1.20 ± 0.17	1.07 ± 0.11	0.528
Item-2: Sleep latency	1.85 ± 0.42	0.95 ± 0.24	0.077	0.78 ± 0.38	0.73 ± 0.25	0.911	−1.22 ± 0.58	−0.23 ± 0.37	0.159	0.98 ± 0.30	1.18 ± 0.28	0.627
Item-3: Sleep duration	1.25 ± 0.22	1.23 ± 0.17	0.935	2.11 ± 0.21	1.36 ± 0.23	0.023	0.78 ± 0.31	0.14 ± 0.27	0.124	2.11 ± 0.11	1.76 ± 0.12	0.034
Item-4: Habitual sleep efficiency	0.50 ± 0.17	0.55 ± 0.22	0.869	0.72 ± 0.28	0.36 ± 0.15	0.270	0.22 ± 0.32	−0.18 ± 0.21	0.301	0.67 ± 0.21	0.51 ± 0.13	0.514
Item-5: Sleep disturbances	1.10 ± 0.36	0.27 ± 0.16	0.047	0.50 ± 0.22	0.41 ± 0.28	0.801	−0.72 ± 0.39	0.14 ± 0.31	0.092	0.38 ± 0.14	0.41 ± 0.17	0.878
Item-6: Use of sleeping medication	0.30 ± 0.21	0.27 ± 0.19	0.923	0.00 ± 0.00	0.00 ± 0.00	—	−0.33 ± 0.23	−0.27 ± 0.19	0.839	0.12 ± 0.09	0.00 ± 0.00	0.187
Item-7: Daytime dysfunction	2.45 ± 0.38	1.91 ± 0.35	0.304	2.72 ± 0.31	2.59 ± 0.31	0.768	0.44 ± 0.52	0.68 ± 0.45	0.732	2.61 ± 0.30	1.91 ± 0.21	0.065
Global PSQI Score	8.70 ± 1.28	6.09 ± 0.84	0.098	8.11 ± 0.82	6.32 ± 0.75	0.113	−0.78 ± 1.60	0.23 ± 1.07	0.605	8.01 ± 0.75	6.84 ± 0.62	0.235
Global score >5	12 (66.67%)	13 (59.09%)	0.870	13 (72.22%)	12 (54.55%)	0.412	—	—	—	—	—	—

Probiotic increased sleep duration and maintained stable sleep quality throughout travel, whereas placebo participants showed transient reductions. Data are mean ± SD or *n* (%); Mann–Whitney *U* test or *χ*^2^ test used for comparisons. PSQI, Pittsburgh Sleep Quality Index; *p* < 0.05 indicates significance.

#### 3.4.2 Anxiety

Anxiety levels, measured using the Generalized Anxiety Disorder Scale (GAD-7), were significantly lower in the probiotic group during travel (*p* = 0.009), at post-travel (*p* = 0.001), and for the change from baseline (*p* = 0.011) (Table 5). The largest between-group difference was observed for the “feeling nervous or on edge” item (*p* = 0.013). Daily assessments showed lower total GAD-7 scores in the probiotic group during travel (*p* < 0.05; Supplementary Figure 2A). Item-level analyses showed that score for item 1 (feelings of nervousness, anxiety, or being on edge) was lower in the probiotic group at Day 2, Day 5, and difference between baseline and Day 5 (*p* < 0.05; Supplementary Figure 2B). Mean GAD-7 scores in both groups remained within the non-clinical range (score <5) throughout the study, indicating the absence of clinically diagnosable anxiety. While these changes occurred within the non-clinical range, they were interpreted as improvements in emotional regulation and stress resilience, rather than treatment of pathological anxiety. This distinction is clinically relevant in preventive contexts such as travel, where mitigation of stress-related symptoms is a primary goal.

**Table 5:** Anxiety levels before, during, and after travel assessed by GAD-7.

Question	Baseline (Day 0)			Post travel (Day 5)			Difference Day 0 and Day 5			During travel (average Days 1–4)		
	Placebo	Probiotic	*p*-value	Placebo	Probiotic	*p*-value	Placebo	Probiotic	*p*-value	Placebo	Probiotic	*p*-value
Item-1: Feeling nervous, anxious or in edge	0.25 ± 0.14	0.55 ± 0.21	0.245	0.89 ± 0.28	0.09 ± 0.09	0.013	0.61 ± 0.32	−0.45 ± 0.23	0.012	0.71 ± 0.22	0.24 ± 0.09	0.064
Item-2: Not being able to stop or control worrying	0.25 ± 0.14	0.00 ± 0.00	0.096	0.00 ± 0.00	0.00 ± 0.00	—	−0.28 ± 0.16	0.00 ± 0.00	0.096	0.03 ± 0.03	0.00 ± 0.00	0.331
Item-3: Worrying too much about different things	0.00 ± 0.00	0.00 ± 0.00	—	0.22 ± 0.15	0.00 ± 0.00	0.163	0.22 ± 0.15	0.00 ± 0.00	0.163	0.03 ± 0.03	0.03 ± 0.02	0.867
Item-4: Difficulty relaxing	0.00 ± 0.00	0.00 ± 0.00	—	0.33 ± 0.18	0.00 ± 0.00	0.083	0.33 ± 0.18	0.00 ± 0.00	0.083	0.18 ± 0.10	0.03 ± 0.02	0.182
Item-5: Being so restless that it is hard to sit still	0.00 ± 0.00	0.00 ± 0.00	—	0.28 ± 0.19	0.00 ± 0.00	0.172	0.28 ± 0.19	0.00 ± 0.00	0.172	0.21 ± 0.14	0.00 ± 0.00	0.164
Item-6: Becoming easily annoyed or irritable	0.20 ± 0.12	0.27 ± 0.16	0.720	0.00 ± 0.00	0.00 ± 0.00	—	−0.22 ± 0.13	−0.27 ± 0.16	0.810	0.15 ± 0.09	0.01 ± 0.01	0.140
Item-7: Feeling afraid as if something terrible might happen	0.00 ± 0.00	0.00 ± 0.00	—	0.00 ± 0.00	0.00 ± 0.00	—	0.00 ± 0.00	0.00 ± 0.00	—	0.00 ± 0.00	0.00 ± 0.00	—
Total score	0.70 ± 0.36	0.82 ± 0.24	0.786	1.72 ± 0.41	0.09 ± 0.09	0.001	0.94 ± 0.55	−0.73 ± 0.27	0.011	1.31 ± 0.33	0.32 ± 0.10	0.009

Participants receiving probiotics reported significantly lower anxiety scores than placebo during and after travel, indicating reduced stress and better emotional stability. Data are mean ± SD; analyzed using Mann–Whitney *U* test. GAD-7, Generalized Anxiety Disorder 7-item scale; *p* < 0.05 significant.

#### 3.4.3 Well-being

Well-being, assessed by the WHO-5 index, was higher in the probiotic group post-travel (*p* = 0.041) and for the change from baseline (*p* = 0.046) (Table 6). Significant improvements were observed in items related to vitality (*p* = 0.044), restful waking (*p* = 0.034), and daily interest (*p* = 0.044). Notably, these enhancements appeared from Day 2 onward and persisted after return, reflecting sustained psychological benefits. Daily analyses confirmed higher scores in the probiotic group at Day 2 and Day 5 (*p* < 0.05; Supplementary Figure 3A). Item-level analyses showed higher scores for the probiotic group for item 3 (feeling active and vigorous) at Day 5 (*p* = 0.044; Supplementary Figure 3B), for item 4 (waking up fresh and rested) at Day 2 and Day 5 (*p* < 0.05; Supplementary Figure 3C), and for item 5 (daily life filled with things of interest) at Day 2 (*p* = 0.020; Supplementary Figure 3D). Notably, the relative increase in WHO-5 score in the probiotic group exceeded 10%, a threshold commonly regarded as clinically meaningful in both clinical and population-based studies. Improvements were particularly evident in items related to vitality, restful waking, and daily interest, suggesting enhanced daily functioning and subjective well-being rather than transient mood fluctuation. These findings indicated that probiotic supplementation conferred a perceptible improvement in overall well-being during short-term travel.

**Table 6:** WHO-5 well-being index before, during, and after travel.

Question	Baseline (Day 0)			Post travel (Day 5)			Difference Day 0 and Day 5			During travel (average Days 1–4)		
	Placebo	Probiotic	*p*-value	Placebo	Probiotic	*p*-value	Placebo	Probiotic	*p*-value	Placebo	Probiotic	*p*-value
Item-1: I have felt cheerful and in good spirits	2.75 ± 0.32	3.00 ± 0.23	0.525	2.72 ± 0.32	3.45 ± 0.21	0.065	0.00 ± 0.36	0.45 ± 0.28	0.326	2.82 ± 0.31	3.35 ± 0.21	0.169
Item-2: I have felt calm and relaxed	2.55 ± 0.29	2.68 ± 0.25	0.735	2.56 ± 0.35	3.32 ± 0.22	0.079	0.00 ± 0.30	0.64 ± 0.29	0.138	2.62 ± 0.32	3.24 ± 0.22	0.123
Item-3: I have felt active and vigorous	2.95 ± 0.27	2.77 ± 0.25	0.633	2.61 ± 0.36	3.50 ± 0.22	0.044	−0.33 ± 0.33	0.73 ± 0.22	0.013	2.69 ± 0.31	3.36 ± 0.21	0.083
Item-4: I woke up feeling fresh and rested	2.65 ± 0.31	2.59 ± 0.27	0.886	2.22 ± 0.36	3.18 ± 0.24	0.034	−0.44 ± 0.38	0.59 ± 0.36	0.055	2.53 ± 0.32	3.31 ± 0.20	0.048
Item-5: My daily life has been filled with things that interest me	2.45 ± 0.29	2.77 ± 0.26	0.418	2.61 ± 0.37	3.41 ± 0.22	0.077	0.11 ± 0.28	0.64 ± 0.34	0.239	2.58 ± 0.33	3.40 ± 0.21	0.044
Total score	53.40 ± 5.41	55.27 ± 4.05	0.783	50.89 ± 6.73	67.45 ± 3.81	0.041	−2.67 ± 5.66	12.18 ± 4.41	0.046	53.00 ± 6.19	66.64 ± 4.00	0.074

Participants receiving probiotics showed enhanced subjective well-being, particularly in measures of vitality and restful waking, compared with placebo. Higher scores indicate better perceived well-being. Data are mean ± SD; Mann–Whitney *U* test applied. WHO-5, World Health Organization Well-Being Index; *p* < 0.05 significant.

### 3.5 Gut microbiota composition

#### 3.5.1 Alpha diversity

**Figure 2:**
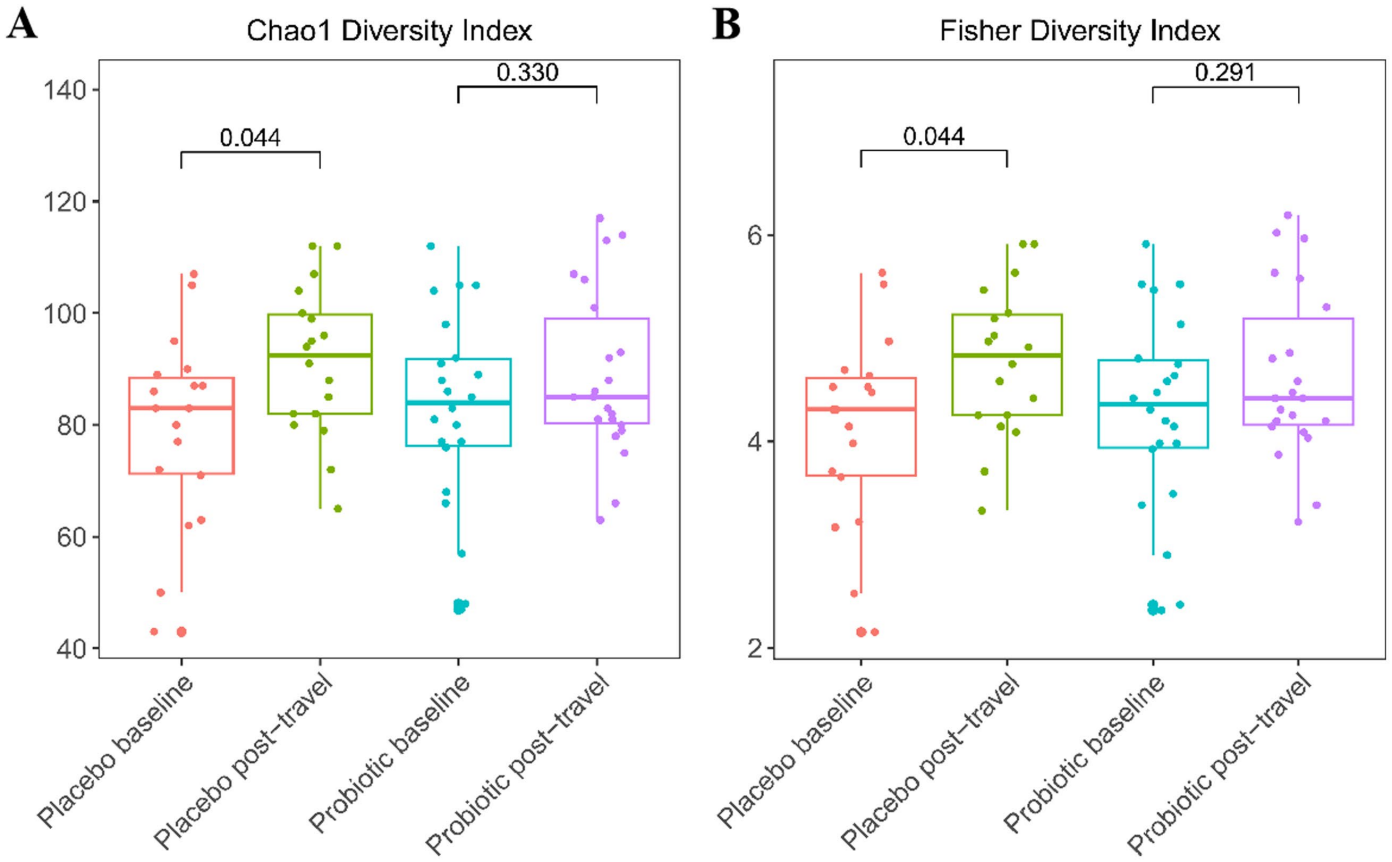
Alpha diversity indices Chao1 **(A)** and Fisher **(B)** before and after travel. Boxplots depict microbial diversity within each group; line inside the box represents the median; whiskers represent the lowest and highest values within the interquartile range (IQR). Diversity remained stable in the probiotic group but increased significantly in placebo (*p* = 0.044), indicating transient dysbiosis. The Mann–Whitney *U* test was used for group comparisons; *p* < 0.05 significant.

Alpha diversity remained stable in the probiotic group but increased significantly in the placebo group between baseline and post-travel (Chao1 and Fisher indices, both *p* = 0.044; Figure 2). The apparent rise in placebo diversity likely reflected transient proliferation of opportunistic taxa rather than beneficial enrichment. This pattern suggests that the increase in diversity observed in the placebo group likely reflected transient microbial disruption, whereas probiotics helped maintain a more stable microbial ecosystem.

#### 3.5.2 Beta diversity

**Figure 3:**
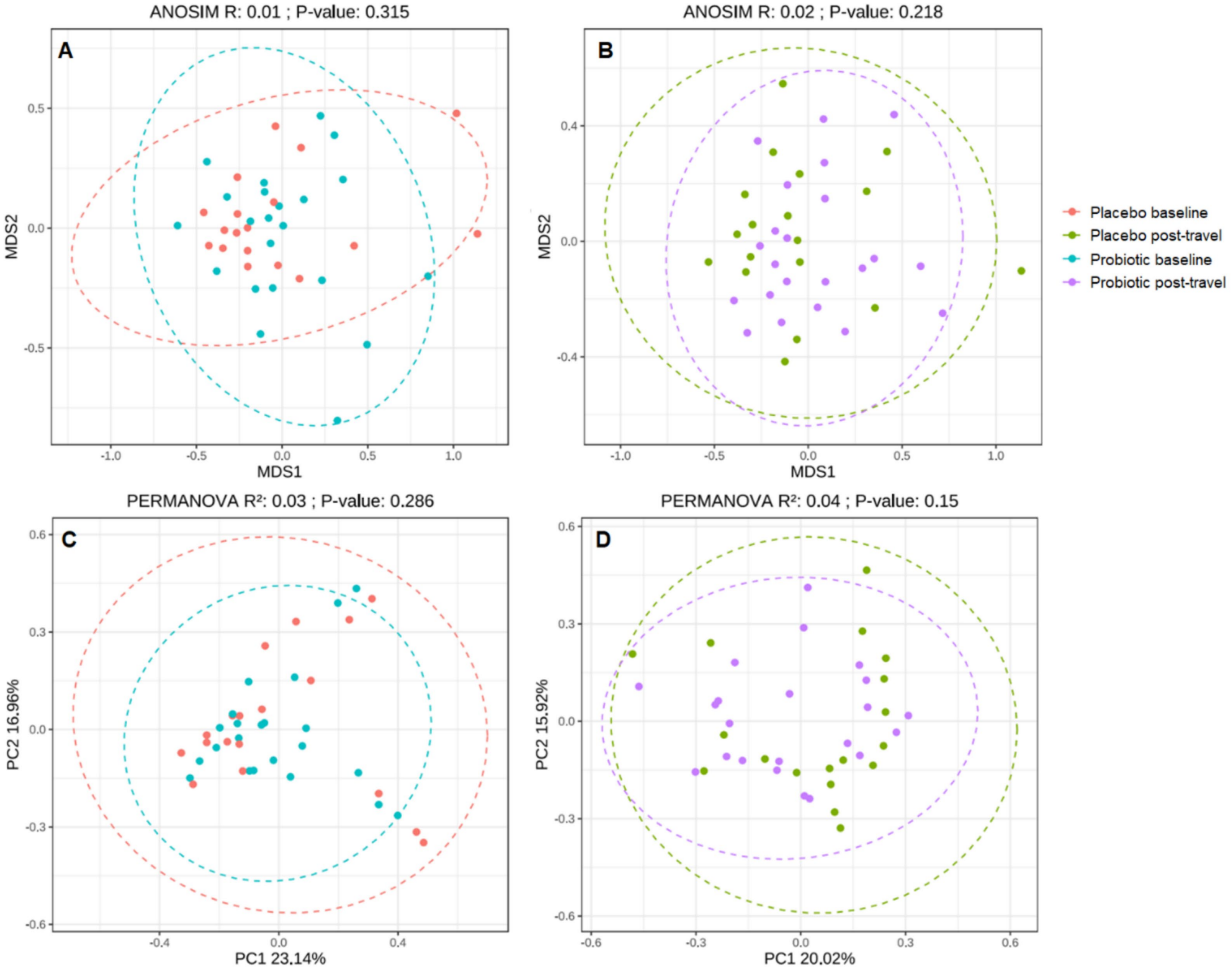
Beta diversity analysis of gut microbial community structure. NMDS **(A,B)** and PCoA **(C,D)** plots based on Bray–Curtis distance show overlapping clusters between probiotic and placebo groups. No significant differences detected by PERMANOVA (*p* > 0.05). Overall microbial community structure remained stable in both groups, suggesting resilience to short-term travel stress. NMDS, non-metric multidimensional scaling; PCoA, principal coordinate analysis.

Beta diversity analyses (Bray–Curtis index) visualized by non-metric multidimensional scaling (NMDS) and principal coordinate analysis (PCoA) showed overlapping clusters between groups (Figure 3). Statistical testing (ANOSIM, PERMANOVA) revealed no significant overall compositional divergence (*p* > 0.05), suggesting general microbiota stability over the short travel period. Overall, the absence of marked beta diversity shifts indicates that short-term travel induced subtle rather than wholesale microbiota restructuring.

#### 3.5.3 Taxonomic profiles

**Figure 4:**
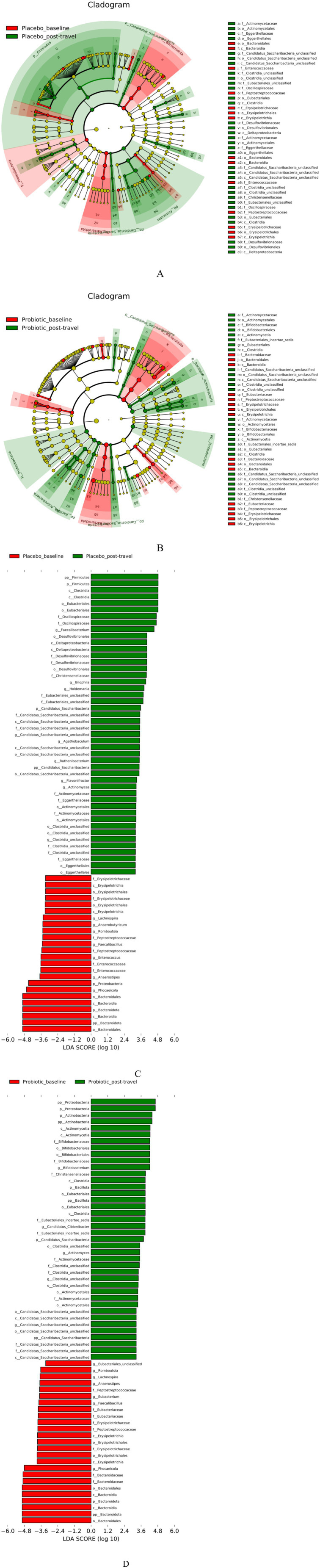
LEfSe-based identification of differentially abundant taxa before and after travel. Cladogram and LDA bar plots highlight taxa enriched in the placebo **(A,C)** and probiotics **(B,D)** groups. *Bifidobacterium* increased in the probiotic group, while *Bilophila* and *Flavonifractor* expanded in placebo. LDA threshold >2.0, *p* < 0.05. Probiotic intake selectively promoted beneficial genera and prevented enrichment of opportunistic taxa associated with inflammation. LDA, linear discriminant analysis; LEfSe, linear discriminant analysis effect size.

Linear discriminant analysis effect size (LEfSe) identified taxa that discriminated between groups (Figure 4). At baseline, both groups were enriched in SCFA-producing genera such as *Phocaeicola*, *Lachnospira*, *Anaerostipes*, *Romboutsia*, and *Faecalibacillus*. The placebo group exhibited slightly higher *Anaerobutyricum* and *Enterococcus*, whereas *Eubacterium* was more abundant in the probiotic group. Post-travel, increases in *Actinomyces* were observed in both groups. However, *Flavonifractor*, *Holdemania*, and *Bilophila*, taxa linked to inflammation and bile-acid dysregulation, were enriched only in placebo participants. The probiotic group instead showed increased *Bifidobacterium*, consistent with the administered strains. Our results indicate that probiotics selectively enriched beneficial *Bifidobacterium* while limiting the emergence of taxa associated with inflammation and dysbiosis.

#### 3.5.4 Targeted quantification of *Bifidobacterium* species

To complement the LEfSe analysis, which indicated enrichment of *Bifidobacterium* in the probiotic group, we conducted targeted quantitative PCR to examine the dynamics of major *Bifidobacterium* species during short-term travel. Because the administered probiotic formulation contained *B. longum* subsp. *infantis* M-63, *B. breve* M-16V, and *B. longum* BB536, these taxa were specifically analyzed alongside other prevalent commensal *Bifidobacterium* species.

**Figure 5:**
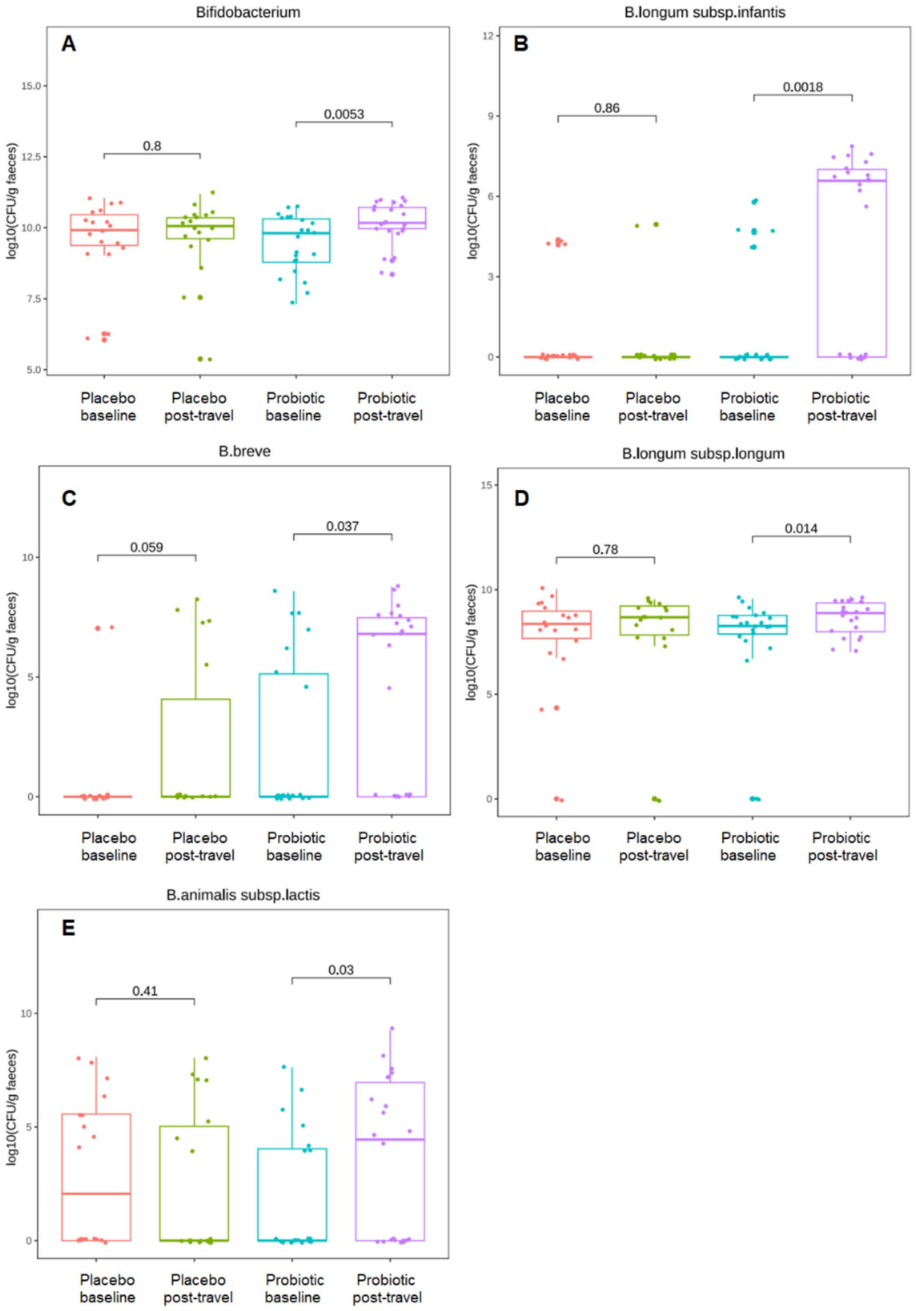
Quantitative profiling of fecal *Bifidobacterium* species before and after short-term travel. Overall abundance of *Bifidobacterium* increased in the probiotic group post travel while the placebo group did not. Values are expressed as log₁₀ CFU/g feces determined by qPCR for: **(A)** total *Bifidobacterium*, **(B)** *B. longum* subsp. *infantis*, **(C)** *B. breve*, **(D)** *B. longum* subsp. *longum*, **(E)** *B. animalis* subsp. *lactis*. Boxes denote interquartile range with median lines; whiskers indicate minima and maxima. Comparisons were performed using the Mann–Whitney *U* test.

At the genus level, the overall abundance of *Bifidobacterium* increased significantly in the probiotic group after travel compared with baseline (*p* = 0.005; Figure 5A). Among the administered strains, *B. longum* subsp. *infantis* (*p* < 0.001; Figure 5B), *B. breve* (*p* = 0.037; Figure 5C), and *B. longum* subsp. *longum* (*p* = 0.014; Figure 5D) all exhibited marked post-travel increases. An additional rise was observed in *B. animalis* subsp. *lactis* (*p* = 0.030; Figure 5E), a species not included in the probiotic preparation. These trends were not observed in the placebo group. These results confirm successful colonization or enrichment of administered probiotic strains and support their role in maintaining microbial balance during travel.

### 3.6 Fecal secretory immunoglobulin

**Figure 6:**
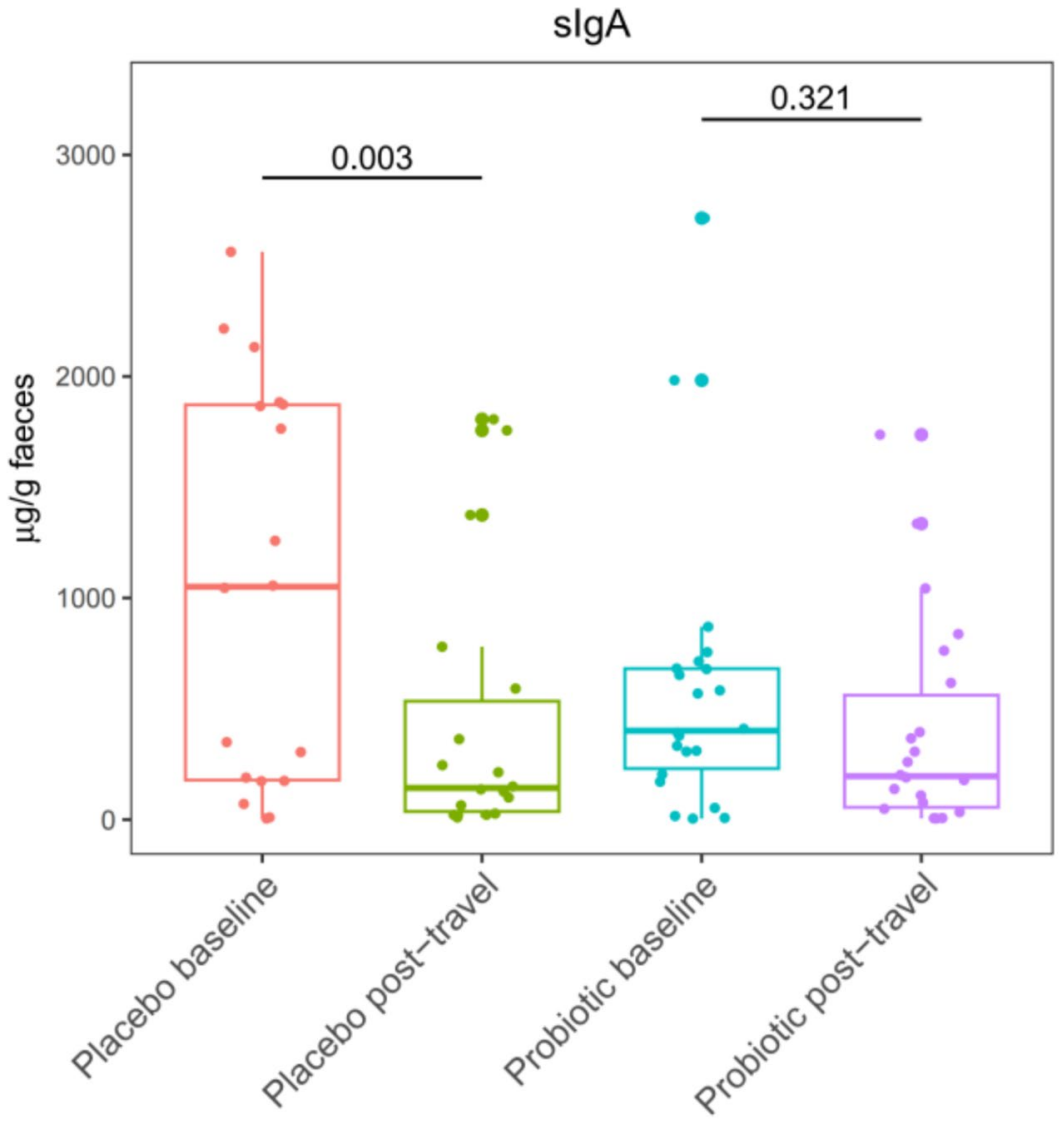
Fecal secretory immunoglobulin A (sIgA) concentrations before and after short-term travel. The probiotic group showed a stable sIgA level while a decrease was observed for the placebo group over time. Boxplots display median values (horizontal lines), interquartile ranges (boxes), and whiskers extending to 1.5× the interquartile range. Comparisons were performed using the Mann–Whitney *U* test.

Fecal sIgA was measured to evaluate mucosal immune responses associated with short-term travel (Figure 6). Baseline sIgA concentrations were comparable between groups. After travel, a significant reduction in sIgA was observed in the placebo group (*p* = 0.003), whereas levels in the probiotic group remained stable, indicating preserved mucosal immune function. Overall, probiotic supplementation preserved mucosal immune function during travel, as evidenced by the maintenance of fecal sIgA levels.

### 3.7 Fecal antibiotic resistance genes

The prevalence of key antibiotic resistance genes (ARGs) was assessed to evaluate whether probiotic supplementation influenced antimicrobial resistance profiles during travel (Table 7). Before travel, *blaSHV* and *tetA* were detected in all participants across both groups. This pattern persisted in the placebo group after travel, whereas the probiotic group showed reduced detection frequencies, with *blaSHV* present in 86.4% and *tetA* in 95.5% of participants post-travel. The *mefE* gene, encoding a macrolide efflux pump, was initially detected in one participant per group but increased to 100% in the placebo group and only 90.9% in the probiotic group after travel. Although within-group changes were not statistically significant, the overall trend suggested that probiotic administration attenuated the travel-associated increase in ARG prevalence. While these findings suggest that probiotics may attenuate travel-associated increases in antibiotic resistance gene carriage. It should be noted that ARG detection in this study was based on conventional PCR and band-intensity assessment, which provides a semi-quantitative measure of gene presence rather than absolute gene copy number. Therefore, the observed differences in detection frequency between groups should be interpreted as indicative of relative ARG prevalence trends rather than precise changes in ARG load per gram of feces. Quantitative approaches such as qPCR or metagenomic resistome profiling will be required in future studies to accurately quantify ARG abundance and evaluate correlations with specific microbial taxa.

**Table 7:** Detection of fecal antibiotic resistance genes (ARGs) before and after short-term travel.

Antibiotic	Placebo			Probiotic		
	Baseline (Day 0)	Post travel (Day 5)	*p*-value	Baseline (Day 0)	Post travel (Day 5)	*p*-value
*blaSHV*			1.000			0.248
Negative	0 (0.0)	0 (0.0)		0 (0.0)	3 (13.6)	
Positive	18 (100.0)	18 (100.0)		22 (100.0)	19 (86.4)	
*mefE*			1.000			1.000
Negative	1 (5.6)	0 (0.0)		1 (4.5)	2 (9.1)	
Positive	17 (94.4)	18 (100.0)		21 (95.5)	20 (90.9)	
*TetA*			1.000			1.000
Negative	0 (0.0)	0 (0.0)		0 (0.0)	1 (4.5)	
Positive	18 (100.0)	18 (100.0)		22 (100.0)	21 (95.5)	

No significant within-group changes for *blaSHV*, *mefE*, and *tetA* genes were observed by McNemar’s test. Values represent the proportion (%) of participants positive for each gene at baseline (Day 0) and after travel (Day 5). ARGs, antibiotic resistance genes; *blaSHV*, β-lactamase gene; *mefE*, macrolide efflux gene; *tetA*, tetracycline resistance gene.

### 3.8 Functional metagenomics

#### 3.8.1 Microbial metabolic pathways

**Figure 7:**
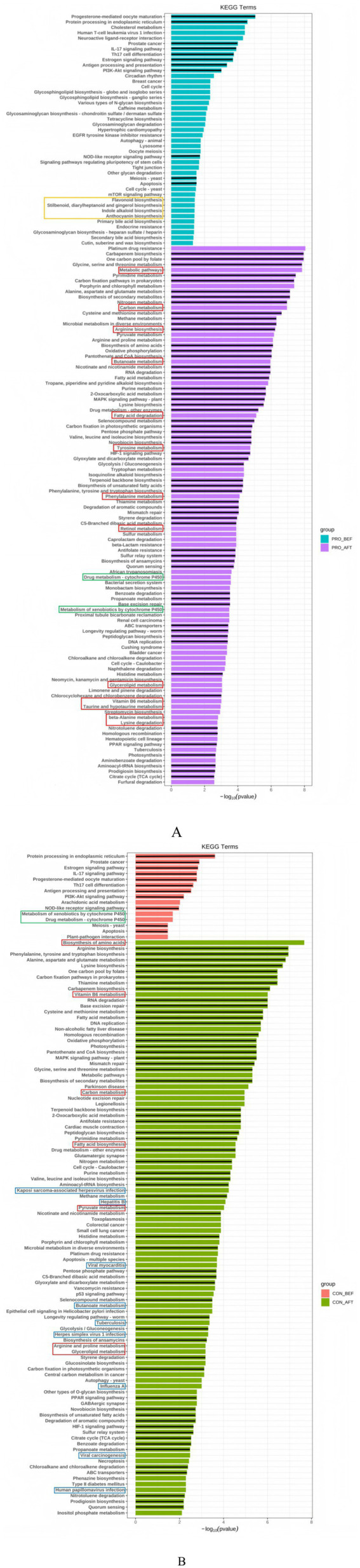
Differential KEGG pathway enrichment in the gut microbiome of participants receiving placebo **(A)** or probiotics **(B)**. Top 100 enriched pathways (*p* < 0.05) per group are shown. Black center lines: shared pathways between groups; red boxes: macronutrient and micronutrient metabolism; blue boxes: viral infection pathways; green boxes: cytochrome P450-related metabolism; yellow boxes: antioxidant biosynthetic pathways. Statistical analysis was performed using the Welch’s *t*-test within STAMP software (*p* < 0.05). KEGG, Kyoto Encyclopedia of Genes and Genomes.

To examine functional shifts within the gut microbiome associated with probiotic supplementation and short-term travel, metagenomic data were mapped to Kyoto Encyclopedia of Genes and Genomes (KEGG) pathways. Before travel, the probiotic group exhibited broader enrichment of metabolic pathways compared with the placebo group, including enhanced biosynthetic routes for antioxidant compounds such as flavonoids, stilbenoids, indole alkaloids, and anthocyanins (Figures 7A,B). In contrast, the placebo group showed a narrower metabolic repertoire characterized by cytochrome P450 (CYP450) activity and limited nutrient biosynthesis pathways.

After travel, the probiotic group demonstrated further upregulation of pathways related to carbohydrate, lipid, protein, amino acid, and vitamin metabolism, indicating an expanded functional capacity for nutrient processing and energy balance. The placebo group also exhibited some enrichment in these categories but with fewer distinct pathways. Pathways associated with viral infection were detected post-travel only in the placebo group, whereas these were absent in participants receiving probiotics. These results indicate that probiotics enhanced metabolic flexibility and functional resilience of the gut microbiome during environmental transition.

#### 3.8.2 Gut vitamin metabolism pathways

**Figure 8:**
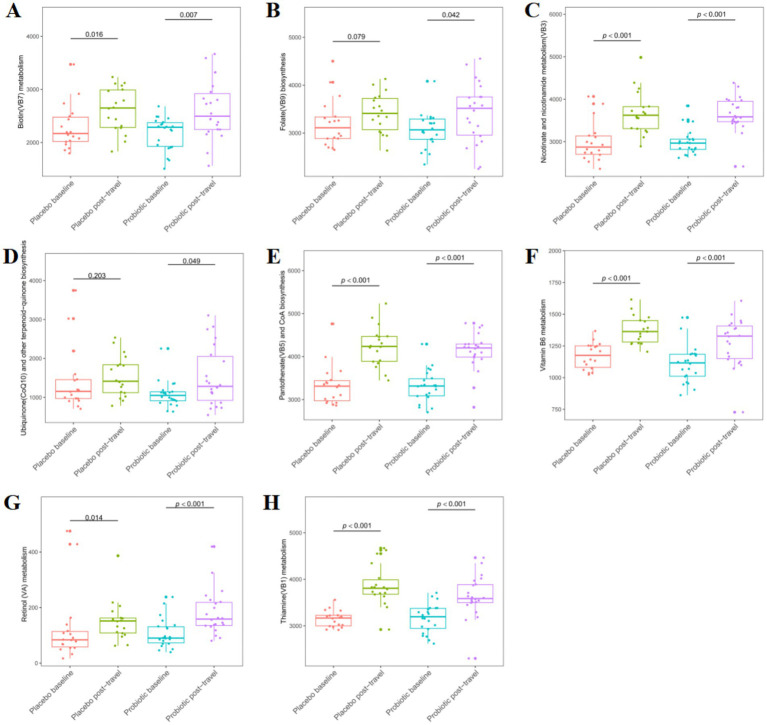
Alterations in vitamin-associated metabolic pathways predicted by KEGG before and after short-term travel. Boxplots display median values (horizontal lines), interquartile ranges (boxes), and whiskers extending to 1.5× the interquartile range for **(A)** biotin (vitamin B7) metabolism, **(B)** folate (vitamin B9) biosynthesis, **(C)** nicotinate and nicotinamide metabolism (vitamin B3), **(D)** ubiquinone and other terpenoid-quinone biosynthesis (Coenzyme Q10 related), **(E)** pantothenate (vitamin B5) and CoA biosynthesis, **(F)** vitamin B6 metabolism, **(G)** retinol metabolism, **(H)** thiamine (vitamin B1) metabolism. Comparisons were performed using the Mann–Whitney *U* test.

Post-travel analysis demonstrated significant upregulation of multiple vitamin metabolism pathways in the probiotic group compared to baseline, including biotin (B7; Figure 8A), folate (B9; Figure 8B), nicotinate and nicotinamide (Figure 8C), ubiquinone and terpenoid-quinone (Figure 8D), pantothenate (B5) and coenzyme A (Figure 8E), vitamin B6 (Figure 8F), retinol (Figure 8G), and thiamine (B1; Figure 8H) (*p* < 0.05). In contrast, the placebo group showed significant upregulation in fewer pathways and with smaller effect sizes, specifically biotin (B7), nicotinate and nicotinamide, pantothenate (B5) and coenzyme A, retinol, and thiamine (B1). Notably, folate (B9), ubiquinone, and terpenoid-quinone pathways were significantly upregulated only in the probiotic group. In summary, probiotic supplementation selectively enhanced microbial vitamin biosynthesis pathways, supporting potential nutritional and immunological benefits during travel.

## 4 Discussion

This randomized, double-blind, placebo-controlled study demonstrated that short-term administration with a *Bifidobacterium* multi-strain formulation effectively stabilized gut microbiota and improved physiological and psychological well-being during international travel. Within a five-day exposure period, probiotics helped preserve microbial diversity, increased *Bifidobacterium* abundance, enhanced vitamin-related metabolic pathways, and reduced the prevalence of potential pathogens and antibiotic resistance genes. Clinically, participants experienced fewer respiratory and systemic discomforts, better stool regularity, improved sleep, reduced anxiety, and higher well-being scores. These findings reinforce and extend the growing evidence that probiotics confer resilience to environmental and lifestyle perturbations. The outcomes also highlight the gut microbiota’s sensitivity to short-term environmental shifts and the capacity of targeted probiotic to restore equilibrium within days.

Although the travel itinerary, duration, and accommodation were standardized for all participants, individual behaviors during travel such as dietary choices, hydration, physical activity, and perceived stress, were not controlled or quantitatively recorded. As a result, inter-individual variability in these lifestyle factors may have contributed to heterogeneity in microbiota composition and symptom reporting. Nevertheless, consistent directional differences observed between groups across multiple clinical and microbiome-related outcomes suggest that the intervention effect was detectable despite this inherent variability.

### 4.1 Linking microbiome stability to subjective well-being during travel

These findings raised an important mechanistic consideration regarding the relationship between improvements in sleep quality, anxiety, and subjective well-being and the microbiome alterations associated with probiotic supplementation. Several observed ecological and functional changes in the gut microbiome provide biologically plausible pathways through which these well-being benefits may be mediated.

First, probiotic supplementation was associated with greater overall microbiota stability, characterized by the maintenance of alpha diversity and the suppression of travel-associated enrichment of taxa such as *Bilophila* and *Flavonifractor*. These genera have been linked to bile acid dysregulation, mucosal inflammation, and stress-sensitive metabolic states. Their selective expansion in the placebo group suggests that short-term travel induced a mild but unfavorable ecological shift, whereas probiotics helped preserve a more balanced microbial environment. Microbiome stability itself has been associated with improved stress tolerance and psychological resilience, particularly during acute environmental perturbations (29).

Second, the taxonomic profile of the probiotic group remained enriched in commensal anaerobes associated with SCFA production, including butyrate- and acetate-producing taxa such as *Faecalibacterium*, *Lachnospira*, and *Anaerostipes*. Butyrate is known to exert anti-inflammatory effects, support intestinal barrier integrity, and modulate neuroimmune signaling via the gut–brain axis (30). Preservation of these functional groups may therefore contribute indirectly to improved sleep and emotional regulation during travel-related stress.

Third, functional metagenomic analysis revealed upregulation of multiple vitamin biosynthesis pathways, including folate (B9), biotin (B7), thiamine (B1), and retinol metabolism, particularly in the probiotic group. Microbially derived B vitamins play essential roles in neurotransmitter synthesis, mitochondrial energy metabolism, and stress-response pathways (31). Enhanced microbial vitamin production may thus support both physical vitality and psychological well-being, consistent with the observed improvements in WHO-5 scores and reduced anxiety symptoms.

In addition, maintenance of fecal secretory IgA levels in the probiotic group suggests preserved mucosal immune homeostasis, which may further contribute to well-being by limiting low-grade inflammation and immune activation during travel. Emerging evidence indicates that immune stability and reduced inflammatory signaling are closely linked to improved mood and sleep quality (32).

Taken together, these findings support a model in which probiotic supplementation promotes microbiome resilience, preserves beneficial metabolic functions, and limits the expansion of stress-associated taxa during short-term travel. Rather than acting through a single metabolite or pathway, the clinical benefits observed, particularly improved subjective well-being, are likely the result of integrated ecological stability and functional robustness of the gut microbiome.

### 4.2 Comparison with previous studies

The present findings were consistent with prior research indicating that travel and dietary transition can rapidly perturb gut microbiota composition and function (33–35). Earlier observational studies showed that international travel, even as brief as 1 week, induces transient dysbiosis characterized by increased microbial diversity and the emergence of opportunistic taxa such as *Bilophila* and *Flavonifractor* (36). Similar shifts were observed in our placebo group, whereas the probiotic group prevented these alterations, underscoring its stabilizing effect.

*Bifidobacterium* species are among the earliest colonizers of the human gut and play central roles in maintaining microbial balance (37–39). Supplementation with *B. longum* BB536 and *B. breve* M-16V has previously been shown to reduce intestinal inflammation and support immune function in both healthy and stressed individuals (40). Our data corroborate these effects, where participants receiving the *Bifidobacterium* combination showed greater microbial stability and sustained sIgA levels compared with placebo.

Clinically, stable bowel patterns and reduction in loose stools in the probiotic group parallels previous meta-analyses reporting that probiotics decrease the risk of travel-related intestinal disturbances and improve bowel regularity (33, 34). Unlike studies focusing primarily on diarrhea prevention (35), this trial observed benefits in gut comfort that substantially affect travel quality.

Moreover, improvements in sleep, anxiety, and well-being align with growing evidence linking probiotics to the gut–brain axis (41, 42). Randomized trials have shown that *Bifidobacterium longum* supplementation alleviates stress-induced anxiety, modulates cortisol levels, and improves subjective sleep quality (43–45). Our findings mirror these results, suggesting that maintenance of microbial stability contributes to neuropsychological resilience during travel-related stress.

### 4.3 Mechanistic considerations

The protective effects observed were likely attributable to multiple complementary mechanisms (46–48). First, the enriched *Bifidobacterium* population likely contributed to colonization resistance against opportunistic bacteria such as *Bilophila* and *Flavonifractor* (36). These genera are associated with bile acid metabolism and mucosal inflammation, and their overgrowth may trigger gastrointestinal discomfort. By maintaining a higher abundance of beneficial anaerobes, probiotics promote a balanced intestinal environment.

Second, functional metagenomic profiling revealed increased activity of vitamin biosynthesis pathways, including folate, biotin, and retinol metabolism. These vitamins play crucial roles in cellular energy metabolism, mucosal repair, and immune regulation (49). Enhanced vitamin production may therefore strengthen epithelial integrity and systemic defense during environmental stress (47, 48).

Third, the preservation of fecal sIgA in the probiotic group supports the hypothesis that probiotics enhance mucosal immunity. Secretory IgA forms a first-line barrier against pathogen adhesion and helps maintain immune tolerance. Stabilization of sIgA levels during travel implies sustained immune surveillance and lower susceptibility to transient infections.

In addition, the psychological benefits likely reflect modulation of the gut–brain axis. *Bifidobacterium* species produce short-chain fatty acids (SCFAs) such as acetate and butyrate, which influence neurotransmitter synthesis and vagal signaling (42). These metabolites can modulate the hypothalamic-pituitary-adrenal (HPA) axis, reducing stress reactivity and improving sleep and mood. Thus, probiotic-mediated microbial stability may help regulate neuroendocrine function, explaining the concurrent improvements in sleep, anxiety, and overall well-being observed in this study.

It should be noted that although improvements in *Bifidobacterium* abundance, microbial vitamin biosynthesis pathways, mucosal immunity (sIgA), and subjective well-being occurred in parallel, direct statistical associations between individual microbial features and clinical endpoints were not formally tested in this study. Consequently, causal inference regarding specific microbial mediators such as whether increases in *Bifidobacterium* directly contributed to reduced anxiety or improved well-being, cannot be established. These findings instead support a model of coordinated microbiome and host responses to probiotic supplementation during travel. Future studies incorporating adequately powered correlation or mediation analyses, alongside integrated multi-omics approaches such as targeted metabolomics, will be essential to elucidate causal pathways linking microbiome-derived metabolites to psychological and immunological outcomes.

### 4.4 Antibiotic resistance gene modulation

An intriguing aspect of this trial is the reduction of antibiotic resistance genes (ARGs) following probiotic supplementation. The *mefE* gene confers resistance to macrolides, lincosamides, and streptogramin B antibiotics (50), whereas *blaSHV* encodes *β*-lactamase enzymes mediating resistance to β-lactam antibiotics (51), and *tetA* contributes to tetracycline resistance (52). The gut microbiota serves as a major reservoir for ARGs, and international travel has been linked to their increased carriage, even without antibiotic use. The observed decrease in *blaSHV*, *mefE*, and *tetA* gene prevalence suggests that probiotics may suppress horizontal gene transfer or reduce niches favorable for resistant taxa. These findings align with the higher abundance of *Bifidobacterium* and the more stable microbial composition observed in the probiotic group. Prior reports found that *Bifidobacterium*-dominant microbiota is less prone to ARG proliferation (53), further supporting their ecological value in maintaining microbial integrity.

CYP450-related metabolism remained active in the probiotic group after travel but was undetectable in the placebo group, despite being present initially. As CYP450 enzymes play key roles in xenobiotic and antibiotic metabolism (54, 55), these findings suggest that probiotic supplementation may have preserved microbial functions linked to detoxification and drug metabolism. This observation aligns with the concurrent reduction of antibiotic resistance genes (*blaSHV*, *mefE*, *tetA*) detected in the probiotic group, implying a functional connection between microbial resilience and resistance gene dynamics.

Collectively, the data indicate that *Bifidobacterium*-based supplementation may contribute to limiting the persistence or expansion of antibiotic resistance determinants during international travel, an effect of potential relevance for mitigating global antimicrobial resistance dissemination.

### 4.5 Strengths and limitations

This study had several strengths. It employed a controlled travel model with standardized itinerary and duration, minimizing variability due to destination and exposure. The integration of clinical, metagenomic, and psychological assessments provided a comprehensive perspective on probiotic effects. Rigorous randomization, double-blind procedures, and full compliance further reinforce data reliability.

The study’s duration was short, reflecting the practical context of brief international trips. While longer-term investigations could extend these insights, the current design effectively captures acute adaptive responses during environmental transition. The participant cohort comprised healthy adults with diverse travel histories, supporting the generalizability of findings to broader populations.

This study has several limitations. The intervention was conducted within a single, structured international travel route (China to Japan), which involved specific dietary, environmental, and circadian challenges; thus, the generalizability of the findings to other travel contexts—such as long-haul intercontinental flights, high-altitude destinations, or regions with distinct endemic microbiomes—remains uncertain. Fecal sampling was limited to two time points, restricting the temporal resolution of microbial dynamics. Furthermore, the assessment of antibiotic resistance genes (ARGs) was based on a semi-quantitative PCR method, which, while effective for determining detection frequency, does not provide the absolute quantification achievable with qPCR.

In addition, the per-protocol analysis included 22 participants in the probiotic group and 18 in the placebo group, a sample size that was powered *a priori* to detect large effects in primary outcomes related to gut microbiota alpha diversity (Cohen’s *d* = 1.0). However, this study was not specifically powered to detect smaller effect sizes that are commonly observed for secondary outcomes, including differential abundance of individual microbial taxa and psychological measures such as anxiety (GAD-7) and well-being (WHO-5). Consequently, some observed effects, particularly those with marginal statistical significance (*p*-values in the range of 0.034–0.046), should be interpreted cautiously, as they may be sensitive to limited statistical power and multiple comparisons. While the consistency of directional effects across related outcomes supports their biological plausibility, confirmatory studies with larger sample sizes and predefined adjustment for multiple testing will be necessary to validate these secondary findings.

Temporal resolution of microbiome sampling represents an additional limitation. Fecal samples were collected only at two time points (pre-travel and post-travel), which restricted the ability to capture dynamic, short-term microbial fluctuations occurring during travel. It is possible that transient alterations in microbial diversity or specific taxa abundance emerged mid-travel and subsequently normalized by Day 5, potentially obscuring differential temporal responses between groups. Mid-travel fecal sampling (e.g., Days 2–3) would provide valuable insight into microbiota kinetics; however, such sampling was not feasible in this study due to practical constraints associated with international travel, including participant burden, and the inability to ensure immediate freezing and continuous cold-chain storage of samples during transit. Future studies may address this limitation by incorporating additional sampling points or utilizing validated room-temperature stabilization methods to better characterize microbiome dynamics during acute environmental transitions.

Although the travel itinerary was standardized, individual-level lifestyle factors during travel, including dietary choices, hydration status, physical activity, and perceived stress, were not comprehensively quantified and may have contributed to inter-individual variability in microbiota and symptom responses. Finally, several outcomes, including sleep quality, anxiety, and well-being, were assessed using self-reported questionnaires; while these instruments are widely validated, they are inherently subject to reporting bias and cannot fully capture objective physiological or neurobiological changes. The absence of mechanistic data, such as metabolomic or cytokine profiles, limits insights into the functional pathways linking the intervention to host outcomes. Future studies would benefit from multiple sampling intervals to define recovery kinetics, quantitative ARG profiling, and the inclusion of multi-omics datasets to elucidate the underlying mechanisms in more diverse populations.

### 4.6 Implications for travel health and microbial resilience

The findings demonstrate that probiotics can enhance biological resilience to environmental, dietary, and psychological stressors encountered during travel. Maintaining gut microbial stability appears pivotal not only for gastrointestinal comfort but also for broader aspects of well-being, including immune competence and mental health.

These results underscore the potential of probiotic interventions as a simple, safe, and practical strategy for travelers. Beyond travel, similar benefits may apply to other transient stress contexts such as shift work, academic pressure, or lifestyle disruption. The preservation of mucosal immunity and reduction of ARG prevalence further suggest a role for probiotics in promoting sustainable microbiome health at the population level.

It should be noted that the findings of this study should be interpreted within the context of a short-duration and structured international trip. The environmental and physiological stressors encountered such as dietary transition and modest circadian disruption, may differ in magnitude and nature from those associated with longer travel durations, long-haul intercontinental flights, high-altitude destinations, or regions with substantially different endemic microbiomes. As a result, the extent to which the observed probiotic-associated benefits generalize to these alternative travel scenarios cannot be determined from the present data. Future studies specifically designed to examine diverse travel contexts will be required to assess the broader applicability of these findings.

## 5 Conclusion

This randomized, double-blind, placebo-controlled trial provided evidence that short-term *Bifidobacterium* supplementation preserves gut microbiota stability, supports mucosal immunity, and promotes physical and psychological well-being during international travel. Participants receiving probiotics experienced reduced respiratory and systemic symptoms, better stool consistency, longer sleep duration, lower anxiety, and enhanced subjective well-being.

Mechanistically, these benefits are linked to sustained *Bifidobacterium* abundance, activation of vitamin biosynthesis pathways, maintenance of sIgA-mediated immunity, and suppression of antibiotic resistance genes. Together, these findings highlight the integral role of the gut microbiome in adapting to environmental change and support the use of probiotics as an accessible approach to enhance resilience during short-term stress.

Further studies incorporating multi-omics profiling will be valuable for defining molecular signatures of probiotic responsiveness and optimizing personalized travel health interventions.

## Data Availability

The datasets presented in this study can be found in online repositories. The names of the repository/repositories and accession number(s) can be found below: NCBI SRA, accession number: PRJNA1417478 (https://www.ncbi.nlm.nih.gov/sra/PRJNA1417478).
