# Supplementary material for: Bifidobacterium supplementation maintains gut microbiota stability and enhances well-being during short-term travel
Source: Front Nutr. 2026 Feb 12;13:1724829. doi: 10.3389/fnut.2026.1724829 (PMC12935942; doi:10.3389/fnut.2026.1724829)
Supplement: Supplementary file 1 [file Supplementary_file_1.docx]

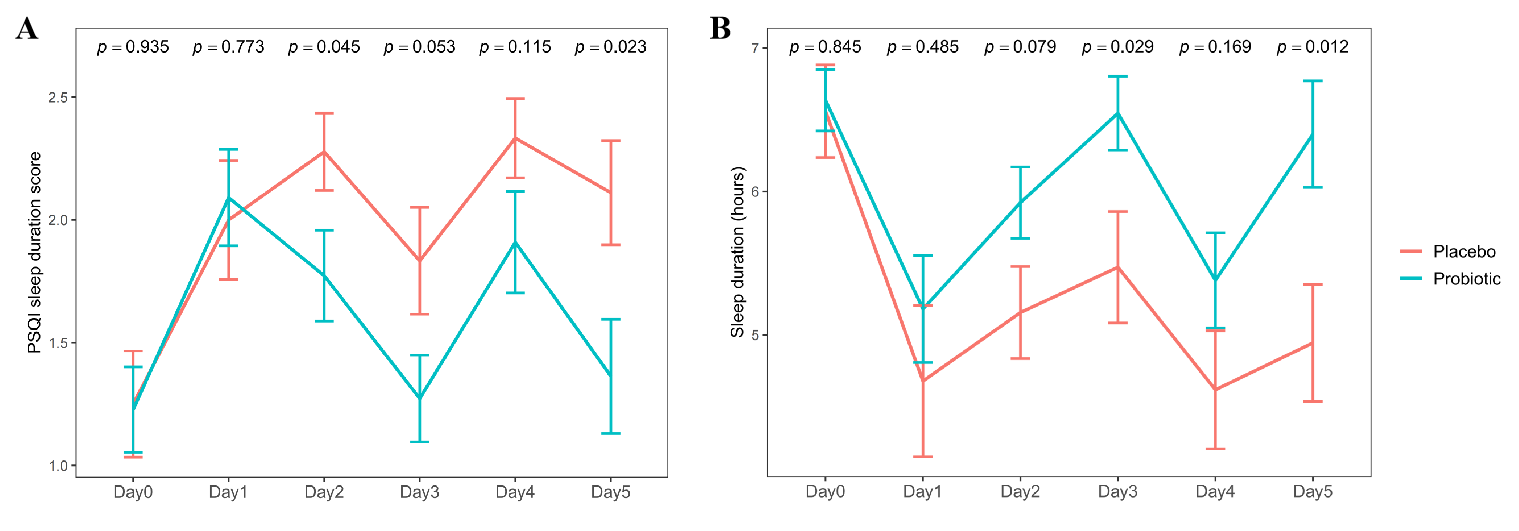
 **Supplementary Figure 1.** Daily PSQI sleep score and actual sleep duration before, during and post travel. Data are mean ± SD; Mann–Whitney U test applied. PSQI = Pittsburgh Sleep Quality Index; *p* < 0.05 significant.


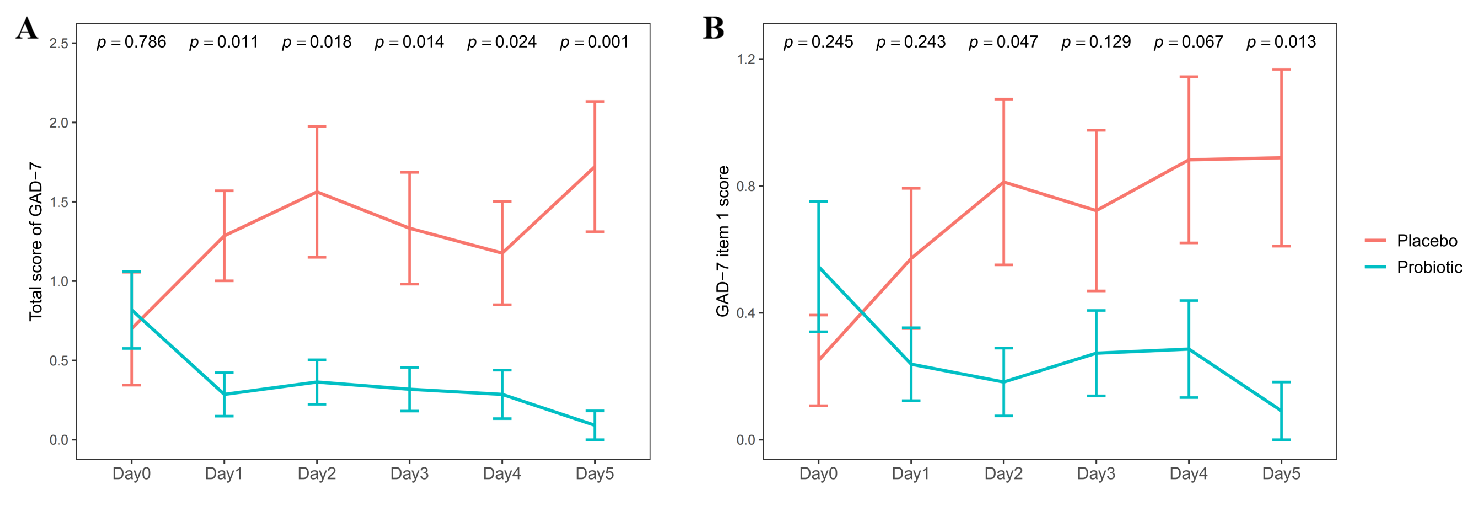


**Supplementary Figure 2.** Daily GAD-7 total, and item-1 scores before, during and post travel. Data are mean ± SD; Mann–Whitney U test applied. GAD-7 = Generalized Anxiety Disorder Scale; *p* < 0.05 significant.


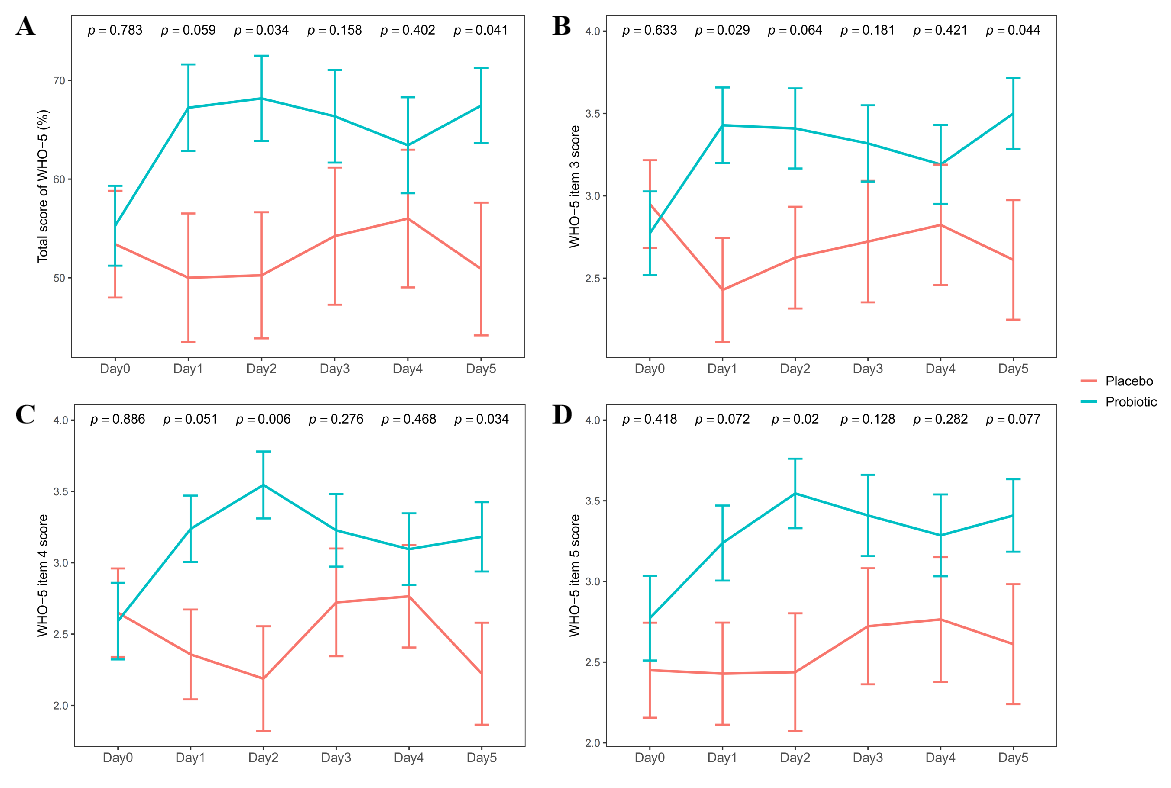


**Supplementary Figure 3.** Daily WHO-5 total, items 3, 4 and 5 scores before, during and post travel. Data are mean ± SD; Mann–Whitney U test applied. WHO-5 = World Health Organization Five Well-Being Index; *p* < 0.05 significant.
